# The temporal topography of the N-Methyl- N-nitrosourea induced photoreceptor degeneration in mouse retina

**DOI:** 10.1038/srep18612

**Published:** 2015-12-21

**Authors:** Ye Tao, Tao Chen, Wei Fang, Guanghua Peng, liqiang Wang, Limin Qin, Bei Liu, Yi Fei Huang

**Affiliations:** 1 Department of Ophthalmology, General Hospital of Chinese PLA, Ophthalmology & Visual Science Key Lab of PLA, Beijing, 100853, PR China; 2 Department of Clinical Aerospace Medicine, Fourth Military Medical University, Xi’an, 710032. PR China; 3 Department of Neurosurgery and Institute for Functional Brain Disorders, Tangdu Hospital, Fourth Military Medical University, Xi’an, 710032, PR China

## Abstract

Retinitis pigmentosa (RP) is a group of inherited neurodegenerative diseases characterized by the progressive photoreceptors apoptosis. The N-Methyl- N-nitrosourea (MNU) is an alkylating toxicant which could induce photoreceptor apoptosis resembling that of the hereditary RP. However, the detailed process pattern of this degeneration remains poorly characterized. We systemically explored the topography of the photoreceptor degeneration in the MNU treated mouse, and related these spatial data with the time-dependent characteristics of retinal pathology. These temporal topographic data delineated sequential scenes of the progressive photoreceptor degeneration in the MNU treated retinas: focal photoreceptors showed different vulnerabilities to the MNU toxicity and displayed a distinctive spatial- and time-dependent progression. Moreover, the positional asymmetry between the retinal quadrants firstly provided instructive information about the unique toxicology properties of the MNU. Further mechanism study suggested that the up-regulation of Bax and Calpain-2, rather than the Caspase-3, should be responsible for the asymmetry in the MNU induced photoreceptor degeneration. Together with the comparative sensitivities to the neurotoxicity of MNU between two photoreceptor populations, these topographic data would facilitate the standardization of analytic parameters related to the MNU induced RP model, and enhance its application in the therapeutic explorations of human RP.

Retinitis pigmentosa (RP) is a group of inherited neurodegenerative diseases characterized by the primary death of photoreceptors, the progressive deterioration of visual fields, and ultimate blindness. The pathological mechanism of RP is not fully understood, neither no satisfactory therapeutic strategy is available: tremendous genetic heterogeneities of RP render it extremely challenging for the accurate genetic diagnosis and specific gene therapies^1^,^2^. Therefore, animal models are essential for furthering our understanding of RP and for developing therapeutic strategies^3^. As an alkylating carcinogen, the N-Methyl- N-nitrosourea (MNU) is proven to be an eligible candidate to selectively introduce photoreceptor death in mammalian retinas^4^,^5^. The MNU-induced photoreceptor death should be attributed to the restriction of deoxyribonucleic acid adduct formation in the nuclei which leads to cell apoptosis. After a single systemic administration, the MNU treated retinas undergo both electrophysiological and morphological alterations similar to the hereditary RP^6^,^7^. Moreover, the damage severity and progression rate of this pharmacological RP model vary with the MNU concentration or application time. These flexibilities largely circumvent the disadvantages in the hereditary RP animal models, such as the unalterable time window for pathologic examination and therapeutic intervention. Thus the MNU induced RP model has been widely utilized in the investigations on human RP^8^,^9^.

Recently, there has been an upsurge of interests in unraveling the pathological mechanism underling the MNU induced photoreceptor degeneration, and several therapeutic trials are based on this highly reproducible model^4^,^10^,^11^,^12^. Although the morphological alterations of this model are well known, several basic issues remains to be addressed. It has been roughly pointed out that the MNU-administrated retina shows time-dependent pathological changes as measured by histological and immunochemical methodologies^4^,^13^. However, the detailed process pattern of this progressive degeneration remains poorly characterized: it is unclear when these pathological changes originate and when they complete fully; neither the original lesion site nor the most resistant zone is exactly located. The time-dependent characteristics are segregated from spatial information and the temporal topography of the MNU induced photoreceptor degeneration is rarely touched. These ambiguities terribly perplex the standardization of the constructive parameters and act as impediments to a broader acceptance of this model to study human RP^5^,^6^.

In the present study, we systemically explored the topography of the photoreceptor degeneration in the MNU administrated mouse, and related these spatial data with the time-dependent retinal pathology. Corresponding with the electroretinography (ERG) and histological results, the positional multi electrodes array (MEA) and flat mount data delineated sequential scenes of the progressive photoreceptor degeneration: focal photoreceptor are comparatively vulnerable to the MNU and exhibited as a distinguished spatial- dependent progression. Moreover, the positional asymmetry among retinal quadrants firstly provided instructive information about the unique toxicology properties of the MNU. Mechanism study found that the Bax and Calpain-2, rather than the Caspase-3, should be responsible for this asymmetry in the MNU induced photoreceptor degeneration. Together with the distinct sensitivities to the MNU between two photoreceptor populations, these topographic results would enrich the knowledge of this neurotoxin, and eventually be instrumental for elucidating the underling mechanism of RP.

## Results

### The topographic morphology of the MNU induced photoreceptor degeneration

**Figure 1:**
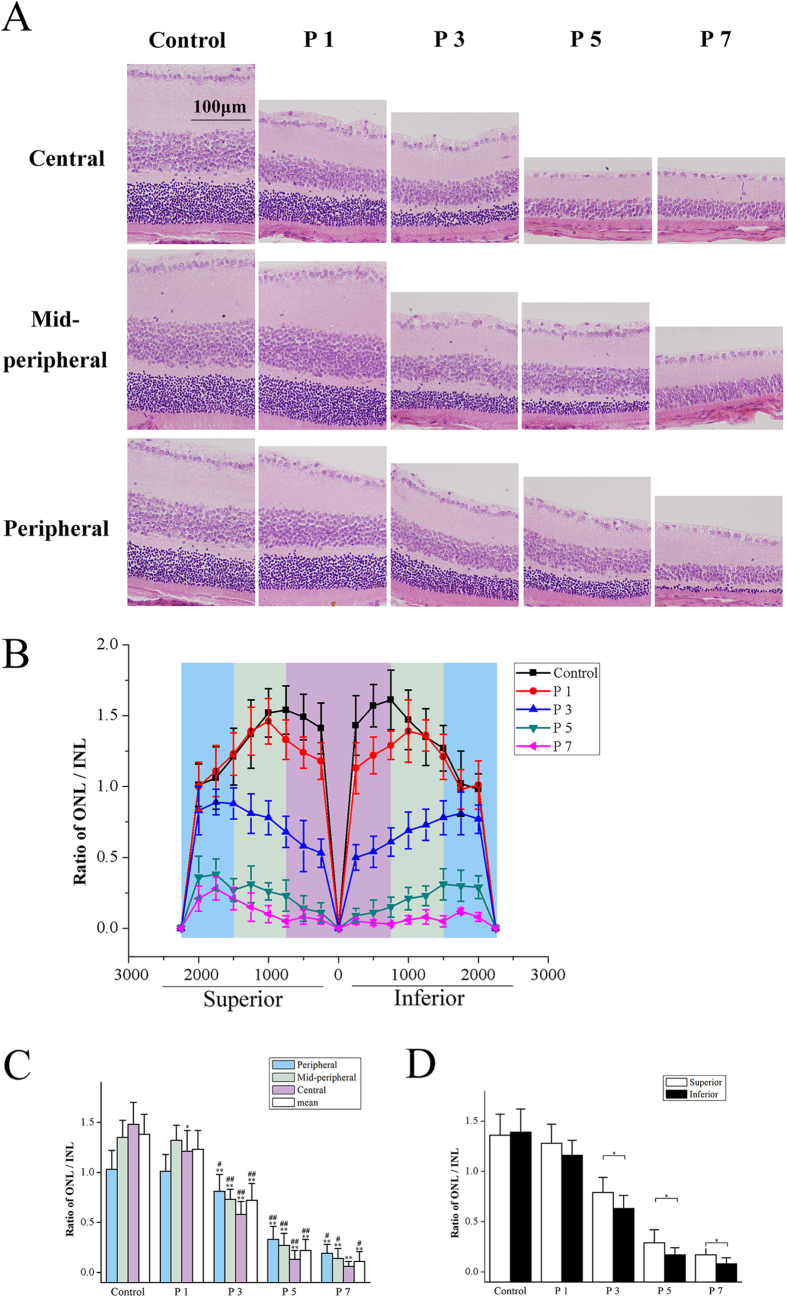
The time-dependent morphological degeneration in the MNU administrated retinas. (**A**) Representative pictures of the central, mid-peripheral, and peripheral retinas along the vertically superior–inferior axis. (**B**) Averaged layer thicknesses at 250 μm intervals were calculated and plotted as morphometric profiles across the vertical meridian. (**C**) Initially at P1, the decrease of the ONL/INL ratio was found in the central region of the MNU administrated retinas, while the ONL/INL ratio of the peripheral and mid- peripheral regions remained unaffected. The decrease of ONL/INL ratio progressed with time, and generalized from the central region to the peripheral and mid- peripheral areas at P3. Subsequently at P5, the ONL of the central region almost disappeared, and the ONL/INL ratio of the mid-peripheral region also remarkably decreased, while the ONL/INL ratio in the peripheral region was retained with relatively ONL larger thickness. By P7, the ONL in the mid-peripheral region also disappeared; the ONL in the peripheral region was left with a minimum of nuclear layers. (**D**) The discrepancy of the degenerated kinetics existed between the superior and inferior hemispheres. The ONL/INL ratio of the inferior hemisphere was statistically smaller than the superior hemisphere respectively at P3, P5, and P7 (ANOVA analysis followed by post-hoc test, n = 10; Fig. C: *P < 0.05, **P < 0.01 for differences compared with controls; ^#^*P* < 0.05, ^##^*P* < 0.01 for differences compared with previous time point group; Fig. D: *P < 0.05, for differences between the superior and inferior hemispheres).

In order to measure the temporal morphology of the MNU induced photoreceptor degeneration, retinal sections were taken along the superior–inferior axis to access the vertical meridian of each hemisphere (Fig. 1A–C). Instantly at the time point of P1, the decrease in the ONL/INL ratio was found in the central region of the MNU administrated retina (*P* < 0.01, P1 vs. control), while the ONL/INL ratio of the peripheral and mid- peripheral areas remained unaffected (*P* > 0.05, P1 vs. control). The decrease of ONL/INL ratio progressed with time, and generalized from the central region to peripheral areas at P3. However, the central photoreceptor was more vulnerable: the ONL/INL ratio of the central region was significantly thinner than the other two regions (*P* < 0.01, central vs. mid- peripheral; P < 0.01, central vs. peripheral). Subsequently at P5, the decrease of the ONL/INL ratio progressed with time and the MNU treated retina underwent massive damages: the ONL/INL ratio in the central region substantially decreased (*P* < 0.01, P5 vs. P3) and the ONL/INL ratio in the mid-peripheral region also decreased (*P* < 0.01, P5 vs. P3). However, the ONL/INL ratio in the peripheral region was relatively retained with larger ONL thickness. By P7, the ONL in the mid-peripheral region also disappeared; the ONL/INL ratio in the peripheral region underwent a continuous decrease and was left with a minimum of nuclear layers (*P* < 0.05, P5 vs. P7). The residual photoreceptors in the peripheral region ultimately disappeared by P9.

Furthermore, the ONL/INL ratio of the superior and inferior retinas was separately examined after MNU administration, indicating different degenerated kinetics between the two hemispheres: the reduction of the ONL/INL ratio was not uniform along the vertical meridian, and substantially greater loss was found in the inferior hemisphere compared with the superior during the whole degeneration. Initially at P1, the ONL/INL ratio of both the superior and inferior hemisphere decreased significantly than that of the controls (*P* < 0.05, P1 vs. control). The ONL/INL ratio of the inferior hemisphere was lower than of the superior, although the difference was not statistically significant (*P* > 0.05, superior vs. inferior). Subsequently at P3, the ONL/INL ratio of the inferior hemisphere was significantly larger than that of the superior hemisphere (*P* < 0.05, superior vs. inferior). Similar results were also found at P5 and P7: the ONL/INL ratio of the inferior hemisphere was consistently smaller than that of the superior hemisphere (*P* < 0.05, superior vs. inferior). This discrepancy in the degenerated kinetics between retinal hemispheres was presented graphically in Fig. 1D, indicating that the superior photoreceptors were more resistant to the toxicity of MNU than these inferior ones.

### The positional degeneration of field potentials after MNU administration

**Figure 2:**
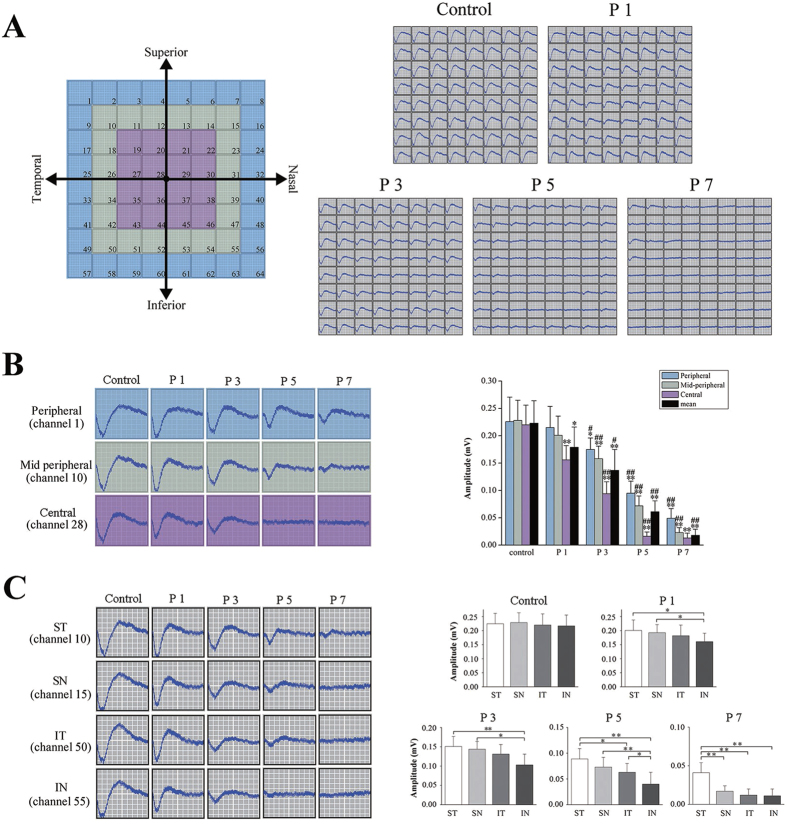
(**A**) Typical negative waveforms of field potential were harvested from the recording patches. (**B**) The central recording channels detected significant decreases in the field potential amplitudes compared with the controls at P1. However, the waveforms of both the peripheral and mid- peripheral regions seemed to be intact. Subsequently at P3, the mean amplitude of field potential was smaller than that of P1. The residual amplitude of the peripheral channels was larger than that of the central and mid- peripheral channels. Subsequently at P5, the field potentials of the central channels were almost undetectable, while the field potentials of the peripheral and mid- peripheral channels were relatively retained. At P7, only minimal responses were detected in the peripheral channels of the ST quadrant. (**C**) The asymmetrical degeneration of the field potentials. The mean amplitude of the IN quadrant was the smallest one among the four quadrants all the time, while the mean amplitude of the ST quadrant was always the largest one. The distribution of field potentials conformed to the following rule: ST > SN > IT > IN (ANOVA analysis followed by post-hoc test, n = 10; Fig. (**B**) *P < 0.05, **P < 0.01 for differences compared with controls; ^#^*P* < 0.05, ^##^*P* < 0.01 for differences compared with previous time point group; Fig. (**C**) *P < 0.05, **P < 0.01 for differences among retinal quadrants).

We performed the MEA recordings at the different time points to examine the functional topography of the MNU induced photoreceptor degeneration. Positional information of focal photoreceptor function could be simultaneously available when the full filed light stimulations were applied. Typical negative waveforms of field potential were harvested in the recording patches (Fig. 2A). No positional difference of the field potential amplitudes was found between the retinal quadrants, neither no positional difference was found between the central, the mid-peripheral and the peripheral regions (*P* > 0.05, Fig. 2B). Immediately at P1, these central recording channels detected a significant decrease of the field potential amplitudes compared with the controls (*P* < 0.01, P1 vs. control). However, the waveforms of both the peripheral and mid- peripheral regions seemed to be intact and no impairment was found (*P* > 0.05, P1 vs. control). Subsequently at P3, the mean field potential amplitude was smaller than P1 (*P* < 0.05, P1 vs. P3). This global decrease was not only be attributed to the progressive decline in the field potential amplitude of the central region (*P* < 0.01, P1 vs. P3), but also due to the reduction of the peripheral and mid- peripheral regions (*P* < 0.05, P1 vs. P3). Moreover, the residual amplitude of the peripheral regions were larger than of the mid- peripheral region (*P* < 0.05, peripheral vs. mid- peripheral). At P5, the negative waveforms in the central region were almost undetectable, whereas the field potentials responses in the peripheral and mid- peripheral regions were relatively retained, albeit with substantially decreased amplitudes (*P* < 0.01, P3 vs. P5). Similar to P3, the residual field potential amplitude of in the peripheral region was larger than the central and mid- peripheral regions at P5 (*P* < 0.01, peripheral vs. central; *P* < 0.05, peripheral vs. mid- peripheral). At the P7, only minimal responses were detected in several peripheral channels which located at the ST quadrant.

The delayed loss of field potential in some peripheral channels implied an asymmetry in the MNU induce photoreceptor degeneration. In fact, the photoreceptor in retinal quadrants showed different susceptibility to the MNU toxicity since P1: the mean amplitude of the IN quadrant was significantly smaller than the ST and SN(*P* < 0.05, IN vs. ST; *P* < 0.05, IN vs. SN, Fig. 2C); It was also lower than that of the IT quadrant, although the difference was not statistically significant. During the following process, the mean amplitude of the IN quadrant was the smallest one among the four quadrants, while the mean amplitude of the ST quadrant was always the largest one; the mean amplitude of the SN quadrant was significantly larger than that of the IT. The amplitude of the retinal quadrants conformed to the following rule: ST > SN > IT > IN. This positional asymmetry between retinal quadrants persisted throughout the whole process of field potential degeneration. At P7, only some residual waveforms with substantially decreased amplitudes were found in the ST quadrant, while these in the other three quadrants disappeared. These vigorous waveforms in the ST quadrant eventually extinguished at P9. The MNU ultimately eliminated all the light induced field potentials of photoreceptors.

### The equilibrium of the RGCs hyperactivities in the MNU administrated retinas

**Figure 3:**
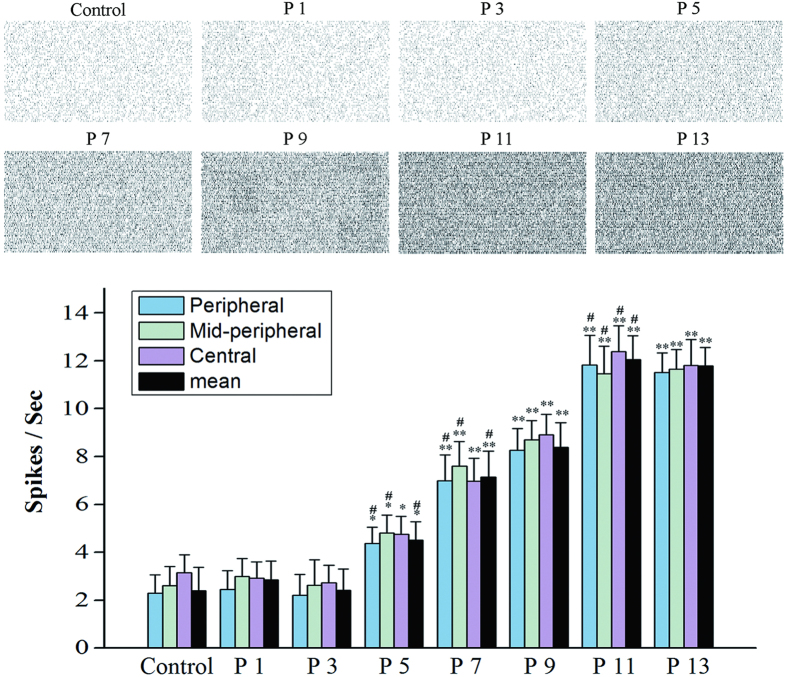
The spontaneous extracellular firing spikes of local RGCs. The spontaneous firing activities of the RGCs were not affected by MNU administration at P1 and P3. However, a significant increase of the spontaneous firing rate was found in the MNU administrated retinas at P5. Subsequently at P7, the spontaneous firing rate in the MNU administrated retinas was even higher. This hyperactivity of RGCs progressed until P12 and remained stable thereafter. (ANOVA analysis followed by post-hoc test, n = 10; *P < 0.05, **P < 0.01 for differences compared with controls; ^#^*P* < 0.05 for differences compared with previous time point group).

The MEA system was used to monitor the spontaneous extracellular firing spikes of local RGCs, providing information about the toxic effects of MNU on the visual signal transmission in the inner retina (Fig. 3). The spontaneous firing activities of the RGCs were not affected by MNU administration initially at P1 and P3 (*P* > 0.05, P1 vs. control; *P* > 0.05, P3 vs. control). However, a significant increase in the spontaneous firing rate was found in the MNU administrated retinas at P5 (*P* < 0.05, P5 vs. control). Subsequently at P7, the spontaneous firing rate in the MNU treated retinas was even higher (*P* < 0.05, P5 vs. P7). This hyperactivity of RGCs progressed until the time point of P11 (*P* < 0.05 P9 vs. P11), and remained stable as long as 45 days thereafter (till P56), despite the PRs has been absolutely ruined by MNU. Unlike the asymmetrical recession of photoreceptor function, the spontaneous activity of local RGCs alternated synchronously in response to the MNU: no significant difference of the firing rate was found between the peripheral, mid peripheral and the central RGCs from P1 to P11. No difference of the firing rate was found between the retinal quadrants throughout the process.

### The progressive ERG deterioration after MNU administration

**Figure 4:**
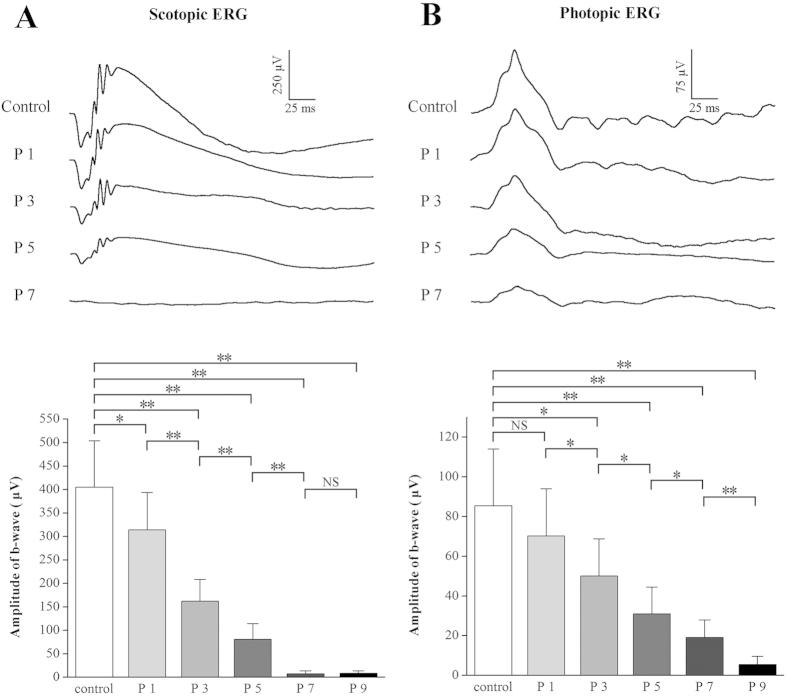
The time-dependent deterioration of both scotopic (**A**) and photopic ERG (**B**) ERGs of the MNU treated mouse. The mean amplitude of the scotopic b- wave decreased significantly in the MNU treated eyes at P1, while the mean amplitude of the photopic b- wave was unaffected. Subsequently at P3, the mean amplitudes of both the scotopic and photopic b- wave decreased significantly in the MNU treated eyes compared with the controls. At P5, the reduction in the mean amplitudes of photopic and scotopic b-wave progressed with time. Furthermore at P7, the scotopic b-waves were undetectable in the MNU treated eyes, whereas a minimum of the b-wave amplitudes were relatively retained in the photopic ERG forms of these eyes. These residual photopic b-waves eventually disappeared by P9 (ANOVA analysis followed by post-hoc test, n = 12; *P < 0.05, **P < 0.01 for differences between groups).

The ERG examination showed a clear time-dependent deterioration of both the photopic and scotopic function after the MNU administration. Figure 4 displayed the representative waveforms of ERG responses in the controls and the MNU-treated eyes. Initially at P1, the mean amplitude of the scotopic b- wave decreased significantly in the MNU treated eyes compared with the controls (*P* < 0.05, P1 vs. control), while the mean amplitude of the photopic b- wave was unaffected (*P* > 0.05, P1 vs. control). Subsequently at P3, the mean amplitudes of both the scotopic and photopic b- wave were reduced significantly in the MNU treated eyes compared with the controls (photopic: *P* < 0.05, P3 vs. control; scotopic: *P* < 0.01, P3 vs. control). At P5, the reduction in the mean amplitudes of photopic and scotopic b-wave progressed with time (photopic: *P* < 0.05, P3 vs. P5; scotopic: *P* < 0.01, P3 vs. P5). Further at P7, the scotopic b-wave was undetectable in the MNU treated eyes (*P* < 0.01, P5 vs. P7), whereas a minimum amplitudes of the photopic b-waves were relatively retained. These residual photopic b-waves eventually disappeared at P9 and the MNU ultimately eliminated all the ERGs of these treated eyes (*P* < 0.01, P7 vs. P9).

### The asymmetry degeneration between photoreceptor populations

**Figure 5:**
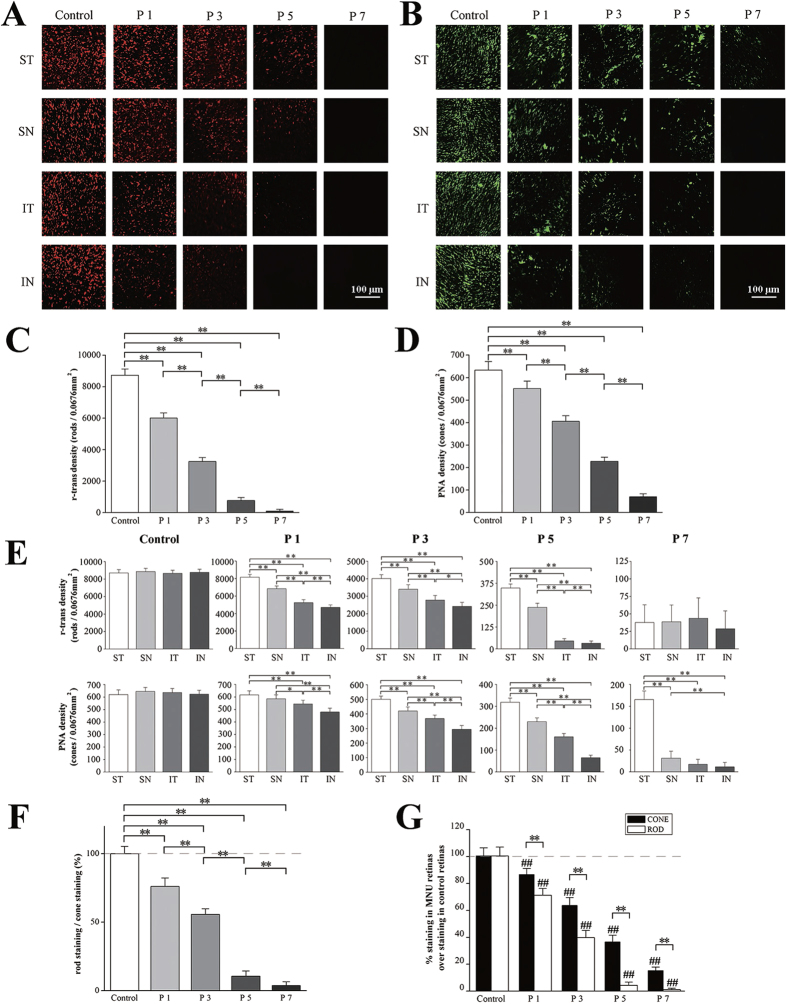
After MNU administration, both the immunostaining of the r-transducin (**A,C**) and the PNA (**B,D**) showed progressive disappearance respectively. (**E**) The most resistant cone staining in the MNU administrated retinas was distributed in the ST quadrant. The cone staining density of the ST quadrant was greater than the other three quadrants at P5 and P7, and it eventually disappeared at P9 while these of other three quadrants disappeared by P7. Similaly, the rod staining density of ST quadrant was also the greatest one, while that of the IN quadrantwas was consistently the smallest one between P1 and P5. Quantification of immunostaining showed that the values of the four quadrants conformed to the following rule: ST > SN > IT > IN. (**F**) The rod immunostaining degenerated disproportionately to the cone immunostaining during the whole degenerated process (**G**). The rod immune staining density decreased by ~30% at P1 compared with the controls, while the cone staining degeneration was less terrible. Later at P3, both the rod and cone staining density reduced significantly: rod by ~57% and cone by ~37%. This inclination progressed till P5, and both staining density underwent substantial reduction. At P7, the rod staining almost vanished, while the cone staining was relatively retained (ANOVA analysis followed by post-hoc test, n = 10; Fig. (**C–F**) *P < 0.05, **P < 0.01 for differences between groups; Fig. (**G**) **P < 0.01 for differences between the rod and cone; ^##^*P* < 0.01 for differences compared with previous time point group).

To study progressive pattern of photoreceptor degeneration over the global retina, we performed the cone and rod immunostaining in these MNU administrated retinas. The r-transducin labeled the rods (Fig. 5A,C) and the PNA labeled the cone outer segments (Fig. 5B,D) both showed progressive degeneration after MNU administration,. However, the rod decreased disproportionately to the cone throughout the whole degenerated process (Fig. 5G). The rod staining density decreased by ~30% at P1 compared with the control (*P* < 0.01, P1 vs. control), while the cone staining density remained relatively preserved and the degeneration was less terrible (by ~14%, *P* < 0.01, P1 vs. control). Later at P3, both the rod and cone staining density reduced significantly: rod reduced by ~57% (*P* < 0.01, P3 vs. control) while cone by ~37% (*P* < 0.01, P3 vs. control). This inclination progressed till P5: a larger drop was found in the rod staining(by ~94%, *P* < 0.01, P5 vs. control) while a lesser drop was found in the cone staining (by ~65%, *P* < 0.01, P5 vs. control). At P7, the rod staining almost vanished (by ~98%, P < 0.01, P7 vs. control), while the cone staining was relatively retained (reduced by ~85%, *P* < 0.01, P7 vs. control). The residual cone staining eventually disappeared at P9. This disproportionate degeneration between the two photoreceptor populations suggested the cones died relatively delayed to the rods. The rods immunostaining was normalized to the cone immunostaining and also demonstrated this disproportion between photoreceptor populations (Fig. 5F).

Moreover, the most resistant cone staining to the MNU insults was also located. Initially at P1, the cone staining density of the ST and SN quadrant was significantly greater than the IT and IN quadrants (Fig. 5E).The cone staining density of the ST quadrant was also higher than that of the SN quadrant,although not statistically significant(*P* > 0.05, ST vs. SN). Subsequently at P3, the cone density of other three quadrants underwent remarkable decrease, but the cone density of the ST quadrant was relatively consolidated. The cone staining density of the ST quadrant remained to be the largest one at P5 and P7, and eventually disappeared at P9, while the cone staining in the other three quadrants disappeared by P7. In parallel to the most resistant cone staining in the ST quadrant, the rod staining density of that region was also the largest one since P1. On the contrary, the rod staining density in the IN quadrantwas was consistently the smallest one. Quantification of rod staining showed that the values of the four quadrants conformed to the following rule: ST > SN > IT > IN. This disequilibrium of rod staining was similar to the previous rules in the MEA tests. Eventually, the rod staining in the IN quadrant vanished by P5, while these in other three quadrants disappeared simultaneously by P7.

**Figure 6:**
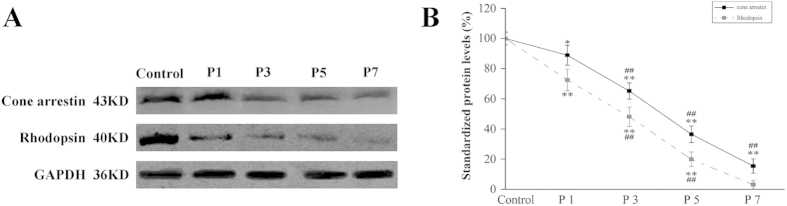
(**A**) Quantification of the photoreceptor-specific proteins levels by western blot analysis. It was found that the expression levels of both the rhodopsin and cone arrestin decreased progressively after P1. However, by the time points of P7, almost all of the rhodopsin extinguished, while a substantially proportion of cone arrestin was relatively preserved. (**B**) The rhodopsin loss was disproportioned to the cone opsin loss during the whole degeneration process: the cone arrestin loss was relatively milder than the rhodopsin loss respectively at P1, P3, (ANOVA analysis followed by post-hoc test, n = 10; *P < 0.05, **P < 0.01 for differences compared with controls; ^#^*P* < 0.05, ^##^*P* < 0.01 for differences compared with previous time point group).

To further confirm the asymmetry degeneration between the two photoreceptor populations, we measured the photoreceptor-specific proteins by western blot analysis (Fig. 6A). Quantification of the photoreceptor-specific proteins levels showed that both the rhodopsin and cone arrestin underwent progressive decrease after P1. However, by the time points of P7, almost all of the rhodopsin extinguished, while a substantially proportion of cone arrestin was relatively retained. Moreover, agreed well with the prior immunostaining results, the western blot document also suggested that the rhodopsin loss was disproportionate to the cone opsin loss throughout the whole process: the expression levels of rhodopsin was lost by about 24%, 47%, 73%, 97% respectively at P1, P3, P5 and P7, while the cone arrestin loss was relatively milder: at P1, P3, P5 and P7 it was lost by 10%, 29%, 60%, and 82% respectively (Fig. 6B).

### The mechanism underling the asymmetric photoreceptor degeneration

**Figure 7:**
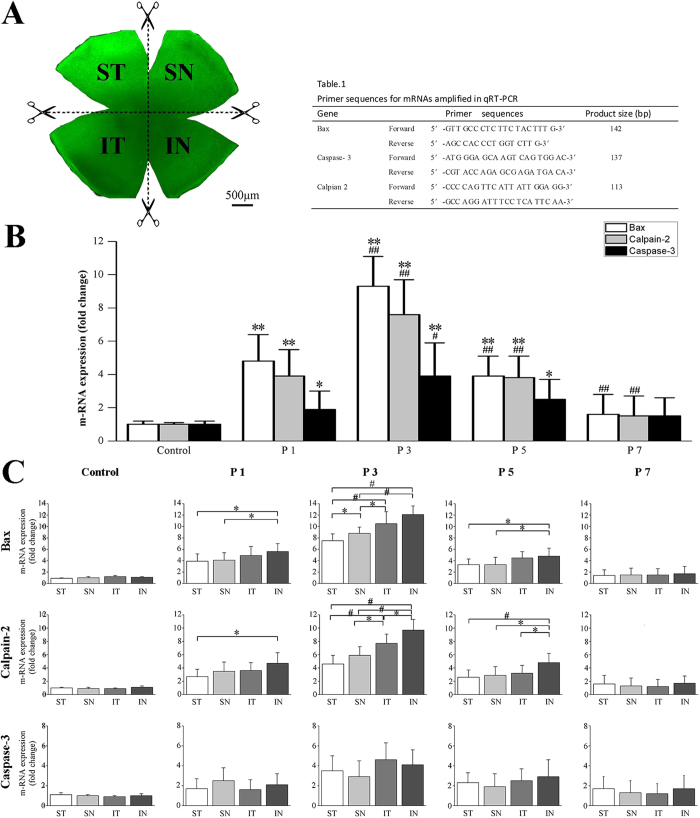
(**A**) The neuroretinal whole mounts were divided into quadrant patches by four incisions at 3, 6, 9 and 12 O’clock. (**B**) The Bax, Calpain-2, Caspase-3 expression were significantly up-regulated at P1 and peaked at P3. At P5, the expression levels of the three markers were down regulated, however, they were significantly higher than the normal control. At P7, the expression levels of the three markers recovered to normal. (**C**) The positional expression levels of these markers were assessed by qRT-PCR. The Bax expression in different retinal quadrants did not changed uniformly since P1. This disequilibrium among retinal quadrants became more obvious at P3 and P5. Similar positional disequilibrium was also found in the whole process of Calpain-2 up-regulation: initially at P1, the expression level in the IN quadrant was higher than the other three quadrants. This positional disequilibrium become more evidently at P3, and persisted throughout the up-regulation till P5. Intriguingly, no positional difference was found between any of the retinal quadrants throughout the whole process of Casepase-3 up-regulation (ANOVA analysis followed by post-hoc test, n = 8; Fig. (**B**) *P < 0.05, **P < 0.01 for differences compared with controls; ^#^P < 0.05, ^##^P < 0.01 for differences compared with previous time point group; Fig. (**C**) *P < 0.05, ^#^*P* < 0.01 for differences between groups).

To explore the possible mechanism underlying the asymmetric photoreceptor degeneration, the expression levels of three specific markers which played significant role in the photoreceptor apoptosis were assessed by qRT-PCR. the Bax, Calpain-2, Caspase-3 expression were significantly up-regulated immediately at P1 (Bax : *P* < 0.01, P1 vs. control; Calpain-2: *P* < 0.01, P1 vs. control; Caspase-3: *P* < 0.05,P1 vs. control; Fig. 7B); The expression levels of these markers peaked at P3 (Bax: *P* < 0.01, P1 vs. P3; Calpain-2: *P* < 0.01, P1 vs. P3; Caspase-3: *P* < 0.05,P1 vs. P3). Subsequently at P5, the expression levels of the three markers were down regulated (Bax: *P* < 0.01, P3 vs. P5; Calpain-2: *P* < 0.01, P3 vs. P5, Caspase-3: *P* > 0.05, P3 vs. P3), however, they were significantly higher than the normal control (Bax: *P* < 0.01, P5 vs. control; Calpain-2: *P* < 0.01, P5 vs. control, Caspase-3: *P* < 0.05, P5 vs. control). At P7, the expression levels of the three markers recovered to normal (*P* > 0.05, P7 vs. control). These findings suggested that the Bax, Calpain-2, Caspase-3 were involved in the procession of MNU induced photoreceptor death.

Furthermore, the positional expression levels of these markers in each retinal quadrants were assessed by qRT-PCR. The Bax expression in different retinal quadrants did not changed uniformly since P1 (*P* < 0.05, IN vs. ST; *P* < 0.05, IN vs. SN, Fig. 7C). This disequilibrium among retinal quadrants became more obvious at P3 (*P* < 0.01, IN vs. ST; *P* < 0.01, IN vs. SN; *P* < 0.01, ST vs. IT; *P* < 0.05, SN vs. IT; *P* < 0.05, SN vs. ST). Subsequently at P5, the positional disequilibrium could also be detected among retinal quadrants (*P* < 0.05, IN vs. ST; *P* < 0.05, IN vs. SN). Similar positional disequilibrium was also found in the whole process of Calpain-2 up-regulation: initially at P1, the expression level in the IN quadrant was higher than other three quadrants (*P* < 0.05, IN vs. ST). This positional disequilibrium become more evidently at P3 (*P* < 0.01, IN vs. ST; *P* < 0.01, IN vs. SN; *P* < 0.01, ST vs. IT; *P* < 0.05, SN vs. IT; *P* < 0.05, IN vs. IT), and persisted throughout the up-regulation till P5 (*P* < 0.01, IN vs. ST; *P* < 0.05, IN vs. SN; *P* < 0.05, IN vs. IT).Intriguingly, no positional difference was found between any of the retinal quadrants throughout the whole process of Casepase-3 up-regulation. These positional findings suggested that the Bax and Calpain-2, rather than the Caspase-3, should be responsible for the asymmetry in the MNU induced photoreceptor degeneration.

## Discussion

Retinitis pigmentosa (RP) is a heterogonous group of non-inflammatory, bilateral, progressive retinal diseases that characterized by the loss of photoreceptors via apoptotic mechanism^1^,^14^. RP animal models are vital for the understandings about underlying mechanisms^15^,^16^. MNU could induce photoreceptor death in a variety of mammalian eyes with active pathological signs which are similar to that in RP patients. Moreover, apoptosis is the primary event and the final common pathway of both human RP and the MNU induced RP models^17^,^18^,^19^. Therefore, this pharmacologically induced RP model has been utilized to identify potential therapeutic strategies for RP, including the neurotrophic factors^20^, antioxidants^21^,^22^,^23^, calcium channel blockers^24^, caspase inhibitors^25^. In the present study, the temporal topography of the photoreceptor degeneration in the MNU administrated mouse was systematically explored. We displayed sequential scenes of the progressive photoreceptor degeneration in terms of structure, function and molecular expression: focal photoreceptor demonstrated different susceptibilities to the MNU toxicity and exhibited as a distinct spatial- and time-dependent degeneration; the positional asymmetry among the retinal quadrants verified an unique pathological property of the MNU induced RP model; distinctive responses of the rod and cone photoreceptors to the MNU poisoning suggested the comparative resistances between the two photoreceptor populations. The time-dependent characteristics were correlated with the spatial topography of the MNU induced photoreceptor degeneration, providing instructive references for the further standardization of this chemically induced RP model.

Consisted with previous reports, positional photoreceptors broke down with different kinetics. The central photoreceptors were more sensitive to the MNU toxicity compared with these peripheral and mid- peripheral ones. A discrepancy in the degenerated kinetics between the superior and inferior hemispheres was found: these superior photoreceptors are more resistant to the MNU toxicity than the inferior ones. Moreover, the start and accomplished time points of the retinopathy varied with the positional sites: the ONL degeneration in the central, mid- peripheral and peripheral regions started at P1, P3, and P3 respectively, while the degeneration accomplished at P5, P7 and P9 respectively.

With the help of the MEA technology, the ensemble photoreceptors in the MNU retina, especially these in peripheral zones, were included in the functional evaluation. Agreed well with the histological results, our MEA data suggested that the topographic visual dysfunction paralleled to the positional photoreceptor death: the MNU induced photoreceptor malfunction originated from the posterior pole and secondarily spread to the peripheral areas. This topographic rule is generally applied to the MNU treated shrews, mice, rats, and hamsters, but not to the primitive primates^26^,^27^,^28^,^29^,^30^. This specie resulted difference should be attributed to the relative number and distribution of rod and cone populations across different mammalian animals^4^,^31^. In the mouse retina, cones are not uniformly distributed and each cone subtype form a gradient across the retina; while the rods, which account for at least 96% of the total photoreceptor, are much more evenly distributed^32^,^33^. Therefore, the above-mentioned topographic property should be considered as an instinctive disadvantage of the rodent MNU induced models because it deviates from the pathology phenotype of human RP, which is characterized by the primary death of the rods and the secondary cone loss in macular: the degeneration progresses form the peripheral to central region, resulting in the gradual constriction of visual fields^1^,^2^.

In greater detail, the MEA recording verified the asymmetry of the functional degeneration among quadrants: the field potential of IN quadrant was the most venerable in MNU administrated retinas, while the field potential of ST quadrant was the most resistant. These temporal topography results displayed where the MNU induced photoreceptor damages originated, how they processed, and when they accomplished. The positional characteristics of the MNU induced photoreceptor degeneration are of significant importance, especially for these explorations aiming at quantifying therapeutic efficiency: the anticipated protective effect should be directly affected by the observed locations, and closely related to the time points when the examination is conducted^34^,^35^. If the experimenter selects different locations between the control and the experimental groups for therapeutic effect analysis, they might possibly mistake the temporarily normal area for the effectively rescued zone. Without the guidance and references of these temporal topography data, these trials would be confronted with higher risk of false error, or even worse, detect an interference of the developmental apoptosis if the administrated retinas are at immature stages^36^,^37^. Future treatments endeavor to restore the vitality of photoreceptors in the MNU administrated retinas should take into account of these temporal topography characteristics, and the appraisal procedures of these therapeutic trials should be timed and located appropriately on the base of them.

In contrast to the asymmetrical recession of photoreceptor function, the spiking activities of the integral RGCs alternated synchronously in response to the MNU toxicity and exhibited as an intriguing hyperactive mode. Our recently pharmacological tests suggested that the gap junctions played a pivotal role in this homogeneous hyperactivity of RGCs^34^. Moreover, possible adjustments in the organization of inner visual signal circuits caused by photoreceptors death should also be responsible for this up-regulation of RGCs activity^7^,^38^. The mismatch between the morphological intactness and the electrophysiological abnormality provides evidences of retinal network plasticity in absence of the presynaptic inputs from photoreceptors^39^,^40^.

Similar to the progressive deterioration of the field potentials and histology, both the scotopic and the photopic ERG degenerated with time after MNU administration. However, the photopic ERG function seemed to be relatively consolidated and retreated in a delayed manner: the onset and accomplished time of the photopic degeneration were both two days later than that of the scotopic degeneration. This discrepancy suggests that the scotopic function is more sensitive to the MNU toxicity than the photopic function, and an asymmetry in the degenerated kinetics might exist. This notion is further validated by the immunofluorescence and western blot analysis: the cones die disproportionately and secondarily to the rods. This pathological characteristic is similar that of RP patients, and should be considered as an advantage of MNU induced RP models. It has been proposed that several potential mechanisms should be attributed to the delayed loss of the cones in the MNU treated retinas: 1) the rods are instinctively more vulnerable to the toxicity of MNU than the cones^17^; 2) the provision of neurotrophic factors which are essential for cone survival has been interrupted as rods die^41^; 3) hyperoxia would introduces oxidative damage to cones in the absence of these metabolically active rods^3^,^42^; Whether the delay of cone degeneration in the present study complies with one or some of these rules remains to be explored. Moreover, in accordance with previous MEA data, both rod and cone immunostaining in the ST quadrant is the most resistant to MNU toxicity among four quadrants. This disequilibrium is also found in the hereditary RP animal models: the superior quadrant with patches of late-surviving cones is the most resistant in the rd1 mouse^43^. In addition, delayed loss of regional PRs has also been reported in the RP patients^44^. Therefore, the present topographic characteristics would justify the utilization of the MNU induced models to study human RP.

The Bax, Calpain-2, Caspase-3 are important factors which contributed to the cell apoptosis^45^,^46^,^47^,^48^. Our qRT-PCR study suggested that these factors were closely related to the progression of MNU induced photoreceptor death. The up-regulated Caspase-3 and Bax expression indicated that the classical apoptotic pathways were switched on by MNU toxicity. On the other hand, the up-regulated Calpain-2, a calcium-dependent cysteine protease, implied that the MNU induced photoreceptor might be closely related to the calcium overload^46^. Furthermore, the positional expression levels of these markers were examined to explore the mechanism underling the topographic characteristics of the MNU induced photoreceptor degeneration. Remarkable positional disequilibrium was found throughout the process of Calpain-2 and Bax up-regulation. Intriguingly, no positional difference was found between any of the retinal quadrants throughout the process of Casepase-3 up-regulation. For the first time, these positional findings verified that the Bax and Calpain-2, rather than the Caspase-3, should be responsible for the asymmetry in the MNU induced photoreceptor degeneration.

In summary, elucidating the temporal topography of the photoreceptor degeneration in these MNU induced animals is crucial for the popularization of this RP model. Our topographic results suggest that the MNU-induced photoreceptor degeneration follow a predictable spatial- and time- dependent pattern. The up-regulation of Bax and Calpain-2, rather than the Caspase-3, should be responsible for the asymmetry in the MNU induced photoreceptor degeneration. The construction of objective therapeutic strategies and related appraisal criterions for the MNU induced photoreceptor degeneration should be based on these data. Improved understandings of the topographic characteristics of the MNU induced photoreceptor degeneration will facilitate the standardization of this pharmacologically induced model, and enhance its use in future therapeutic explorations of human RP.

## Materials and Methods

### Animals and MNU administration

All experiments were conducted in accordance with the ARVO Statements for the Use of Animals in Ophthalmic and Vision Research. All procedures regarding the use and the handling of the animals were conducted as approved by the Institutional Animal Care and Use Committee of the General Hospital of Chinese PLA; All efforts were made to minimize the number of animals used and their suffering. The C57/BL mice with both sexes used in this study were 8 weeks old. All animals were maintained under standard laboratory conditions (room temperature 18 °C to 23 °C, 40% to 65% humidity, 12 h dark/light cycle) with food and water available ad libitum. The MNU (Sigma; St. Louis, MO) was kept at −20 °C in the dark. The MNU solution was dissolved in the physiologic saline containing 0.05% acetic acid just before use. MNU-treated mouse received an intraperitoneal injection of MNU at the dose of 60 mg/kg body weight; Mouse of the control group received an intraperitoneal injection of physiological saline containing 0.05% acetic acid. Over all physiology and food intake of the experimental animals were monitored. No death occurred, and no clinical signs or system symptoms were evident in any of MNU administrated animals during the experiment. Experimental examinations were performed at the time points of P1, P3, P5, or P7 (day post injection).

### ERG recording

The UTAS Visual Diagnostic System with a Big Shot Ganzfeld (LKC Technologies, Gaithersburg, MD, USA) was employed using methods previously described used^6^. Animals were weighed and dark-adapted overnight before ERG analysis. Under dim red light conditions, anesthesia was induced by an intraperitoneal injection of ketamine (80 mg/kg) and chlorpromazine (10 mg/kg). Animals were lightly secured to a stage to ensure a stable position for recording. Platinum circellus record electrodes were placed on each cornea and a reference electrode placed subcutaneously between the eyes. White flashes with the intensity of 0.5 log cd-s/m^2^ were applied for stimulating the scotopic ERGs. Photopic ERG measurements were obtained for flash intensities at the intensity of 1.48 log cd-s/m^2^. Totally 60 Photopic responses and 10 scotopic responses were recorded and averaged for analysis.

### MEA recording

Mice were sacrificed under dim red light and their eyes were enucleated and dissected immediately. The neural retina was gently removed from the pigment epithelium layer of the eye-cup and was placed into the recording chamber, with the ganglion cell layer facing the MEA biochip electrode array. The electrode array used in the present study contains 64 electrodes, which were arranged in 8 × 8 layout as described previously (Alphamed Sciences, Osaka Japan)^34^. During recording, the optic nerve head (ONH) was fixed to the middle of the electrode array. These electrodes were classified into three groups according to their position in the array: the central channels, the mid-peripheral channels, and the peripheral channels (Fig. 2A). Moreover, the global recording field was divided into four quadrants: superior temporal (ST), superior nasal (SN), inferior temporal (IT), and inferior nasal (IN). The retina samples were perfused with oxygenated Ringer’s solution (95% O^2^ and 5% CO^2^) for mammalian retinas. The analog extracellular neuronal signals were detected by the MEA system (MED-64, Alpha med Sciences, Osaka, Japan) and were AC amplified, sampled at 20 kHz and stored in a compatible computer for subsequent off-line software analysis (Neuroexplorer, Nex Technologies, MA, USA) software.

### Stimulus and spike analysis

The responses were elicited following previously described measures^34^. Briefly, the LEDs were driven by a computed stimulator to provide the retina a uniform full field illumination at a mean photonic intensity of 850 mcd·sec/m^2^. Before spike detection, the field potentials were wiped off through a band pass filter (100 Hz to 3000 Hz). The threshold for spike detection was set to four times the standard deviation (SD) of the mean vale of the measured signal for each electrode. These candidate spike waveforms were then sorted by the Offline Sorter. The clusters were firstly identified using a K-mean cluster algorithm, and then manually edited for clustering errors. These units without visual response were categorized as nonresponsive.

### Morphological evaluation by quantitative histology

The eyes were enucleated and a hole was made in the nasal ora serrata with a needle for orientation purposes. The eyecups were immersed in a fixative solution 4% paraformaldehyde (Dulbecco’s PBS; Mediatech, Inc., Herndon, VA) for 6 hours. They were rinsed in PB (phosphate buffer), dehydrated in a graded ethanol series, and embedded in paraffin wax. Five sections (thickness: 4 μm) cut vertically through the ONH of each eye were stained with HE (hematoxylin and eosin) and examined by light microscopy^49^. Image-Pro Plus software (Media Cybernetics, Silver Spring, MD) was used to outline the ONL (outer nuclear layer) and INL (inner nuclear layer). The adjacent thicknesses of the ONL and INL were measured at 250  μm intervals along the vertically superior–inferior axis by a single observer in a masked fashion. Data from three sections (selected randomly form six sections) were averaged for each retina. Averaged Ration of ONL thicknesses to INL thickness at each point were calculated by data form ten retinas from different animals and plotted as a function of eccentricity from the ONH, producing a morphometric profile across the vertical meridian. Similar to the MEA classification, the retina was divided into three regions: central (0–750 μm), mid-peripheral (750–1500 μm), and peripheral (1500–2250 μm) regions.

### Retinal flat mounts preparations

The retinal flatmounts were prepared according to previously described methods^50^. Briefly, the optic nerve bud and its surrounded sclera were cut from the back of the eyecup. Soft touching and gentle pressing by forceps on sclera of the eyecup to help separate the whole neuroretinal layer from the RPE layer, and the eyecup became the neuroretinal flatmounts.

### Immunohistochemistry and density

The flat mounts were rained with PBS and were blocked in 2% normal goat serum, 0.3% Triton X-100 in 1% BSA for 1 hour at room temperature, and then incubated in the polyclonal rabbit anti-rod transducinα (1:200, Sc-389, Santa Cruz Biotechnology, CA) at 4 °C overnight. After extensive washing with buffer, the flat-mounts were incubated in Cy3-conjugated donkey anti-rabbit IgG (1:400, 711-165-152, Jackson ImmunoResearch Laboratories). We also used the peanut agglutinin (PNA) conjugated to Alexa Fluor 488 (1:200, L21409, Invitrogen) to specifically bind with the cone outer segments. The neuroretinal flat mounts were prepared by 4 cuts at 3, 6, 9 and 12 O’clock and were flattened under cover slips with anti-fade vectashield mounting medium (Vector Laboratories, Burlingame, CA) for photographing. Fluorescence in flat mounts was analyzed with the Zeiss LSM 510 META microscope (Zeiss, thornwood, NY) fitted with Axiovision Rel. 4.6 software. The number of cones present within four260 × 260 μm squares located 1 mm superior, temporal, inferior, and nasal to the center of the optic nerve was determined. For quantification, all lighting parameters on the microscope and the camera software were standardized to ensure consistent stable lighting throughout the image capture procedure. A background image of a blank slide was taken for each sample set and was subtracted from the corresponding sample image. The retina was then selected and the integrated density (sum of the pixel values above threshold) of immune staining, as well as the total selected area and its mean labeling intensity (mean value of pixels above threshold) were measured.

### Western blot analysis

The Retinas were collected and homogenized in buffer containing 0.23 mol sucrose, 2 mmol EDTA, 5 mmol Tris–HCl (pH 7.5), and 0.1 mmol phenylmethylsulfonyl fluoride. After centrifugation, aliquot extracts containing equal amounts of protein (20  μg) were electrophoresed, transferred, and probed a polyclonal rabbit anti-cone arrestin (1:1000, sc-67212, Santa Cruz Biotechnology, CA) or polyclonal rabbit anti-rhodopsin (1:1000, sc-15382, Santa Cruz Biotechnology, CA). The membrane was washed thoroughly and then incubated with goat anti-rabbit IgG horseradish peroxidase secondary antibodies (1:3000, sc-2004, Santa Cruz Biotechnology, CA). Visualization of immunoreactive bands was performed using the Chemiluminescence Fluorescence Imaging System (Super Signal ECL kit; West Pico; Pierce, Rockford, IL, USA).

### Quantitative reverse transcription-polymerase chain reaction (qRT-PCR)

Mice were sacrificed and their eyes were enucleated. These eyes were marked at “12 o’clock” with a hot needle which facilitated orientation. The neuroretinal whole mounts were prepared according to previously described methods^50^ and then were divided into quadrant patches by four incisions at 3, 6, 9 and 12 O’clock (Fig. 7A). Total RNA was extracted from these retinal patches with a commercial reagent (Trizol, Gibco Inc., Grand Island, NY), followed by cDNA synthesis using the μ MACS™ DNA Synthesis kit (Miltenyi Biotech GmbH, Bergisch-Gladbach, Germany). Primer sequences are depicted in Table 1 and all primers were quality controlled by sequencing the template on a genetic ABI analyser (Applied Biosystems Inc., Foster City, CA, USA).

The results were normalized with housekeeping gene beta-actin. Reactions were performed with SYBR^R^ Green Master Mix (Bio-Rad Laboratories, Reinach, Switzerland) on a real-time CFX96 Touch PCR detection system (Bio-Rad Laboratories, Reinach, Switzerland). The amplification program consisted of polymerase activation at 95 °C for 5 min and 50 cycles of denaturation at 95 °C for 1 min, annealing and extension at 59 °C for 30 seconds. Duplicate RT-qPCR reactions were performed for each gene to minimize individual tube variability, and an average was taken for each time point. Threshold cycle efficiency corrections were calculated, and melting curves were obtained using cDNA for each individual-gene PCR assay. The relative expression levels were normalized and quantified and using the comparative threshold cycle (Ct): ΔΔCt = ΔCt (sample) − ΔCt (reference gene) (DATA assist Software v2.2, Applied Biosystems).

### Statistical analysis

The values were presented as mean ± standard error of the mean (S.E.M.) unless otherwise specified. The ANOVA analysis followed by Bonferroni’s post-hoc analysis was performed to examine the statistical differences between the control and the MNU treated animals. *P* < 0.05 was considered to be significant. In the MEA recording of RGCs (retinal ganglion cells) activities, the clusters were identified by a K-mean cluster algorithm.

## Additional Information

**How to cite this article**: Tao, Y. *et al.* The temporal topography of the N-Methyl-N-nitrosourea induced photoreceptor degeneration in mouse retina. *Sci. Rep.* **5**, 18612; doi: 10.1038/srep18612 (2015).

## Figures and Tables

**Table 1:** Primer sequences for mRNAs amplified in qRT-PCR.

Gene	Primer sequences	Product size (bp)
Bax	Forward 5′-GTT GCC CTC TTC TAC TTT G-3′	142
	Reverse 5′-AGC CAC CCT GGT CTT G-3′	
Caspase-3	Forward 5′-ATG GGA GCA AGT CAG TGG AC-3′	137
	Reverse 5′-CGT ACC AGA GCG AGA TGA A-3′	
Calpian2	Forward 5′-CCC CAG TTC ATT ATT GGA GG-3′	113
	Reverse 5′-GCC AGG ATT TCC TCA TTC AA-3′	
